# Supplementary material for: Nutritional Characterization of Annual and Perennial Glassworts from the Apulia Region (Italy)
Source: Foods. 2025 Oct 7;14(19):3433. doi: 10.3390/foods14193433 (PMC12524015; doi:10.3390/foods14193433)
Supplement: Supplementary file 1 [file foods-14-03433-s001.zip › foods-3874313-supplementary.pdf]

Table S1. Spearman correlation coefficients among antioxidant assays and total phenols and flavonoids.

Antioxidant assay	Total phenols	Flavonoids
DPPH	0.76***	0.84***
HA[1]-ABTS	0.64***	0.75***
-HA-FRAP	0.61***	0.59***

[1] HA= hydrophilic fraction of antioxidants

***=significant at $p < 0.001$.
